# Prevalence and Determinants of Unintended Pregnancy in Mchinji District, Malawi; Using a Conceptual Hierarchy to Inform Analysis

**DOI:** 10.1371/journal.pone.0165621

**Published:** 2016-10-31

**Authors:** Jennifer Anne Hall, Geraldine Barrett, Tambosi Phiri, Andrew Copas, Address Malata, Judith Stephenson

**Affiliations:** 1 Research Department of Reproductive Health, UCL Institute for Women’s Health, London, United Kingdom; 2 MaiMwana Project, Mchinji, Malawi; 3 Department of Infection & Population Health, UCL Institute of Epidemiology and Health Care, London, United Kingdom; 4 Kamuzu College of Nursing, University of Malawi, Lilongwe, Malawi; Indiana University, UNITED STATES

## Abstract

**Background:**

In 2012 there were around 85 million unintended pregnancies globally. Unintended pregnancies unnecessarily expose women to the risks associated with pregnancy, unsafe abortion and childbirth, thereby contributing to maternal mortality and morbidity. Studies have identified a range of potential determinants of unplanned pregnancy but have used varying methodologies, measures of pregnancy intention and analysis techniques. Consequently there are many contradictions in their findings. Identifying women at risk of unplanned pregnancy is important as this information can be used to help with designing and targeting interventions and developing preventative policies.

**Methods:**

4,244 pregnant women from Mchinji District, Malawi were interviewed at home between March and December 2013. They were asked about their pregnancy intention using the validated Chichewa version of the London Measure of Unplanned Pregnancy, as well as their socio-demographics and obstetric and psychiatric history. A conceptual hierarchical model of the determinants of pregnancy intention was developed and used to inform the analysis. Multiple random effects linear regression was used to explore the ways in which factors determine pregnancy intention leading to the identification of women at risk of unplanned pregnancies.

**Results:**

44.4% of pregnancies were planned. On univariate analyses pregnancy intention was associated with mother and father’s age and education, marital status, number of live children, birth interval, socio-economic status, intimate partner violence and previous depression all at p<0.001. Multiple linear regression analysis found that increasing socio-economic status is associated with increasing pregnancy intention but its effect is mediated through other factors in the model. Socio-demographic factors of importance were marital status, which was the factor in the model that had the largest effect on pregnancy intention, partner’s age and mother’s education level. The effect of mother’s education level was mediated by maternal reproductive characteristics. Previous depression, abuse in the last year or sexual abuse, younger age, increasing number of children and short birth intervals were all associated with lower pregnancy intention having controlled for all other factors in the model. This suggests that women in Mchinji District who are either young, unmarried women having their first pregnancy, or older, married women who have completed their desired family size or recently given birth, or women who have experienced depression, abuse in the last year or sexual abuse are at higher risk of unintended pregnancies.

**Conclusion:**

A simple measure of pregnancy intention with well-established psychometric properties was used to show the distribution of pregnancy planning among women from a poor rural population and to identify those women at higher risk of unintended pregnancy. An analysis informed by a conceptual hierarchical model shed light on the pathways that lead from socio-demographic determinants to pregnancy intention. This information can be used to target family planning services to those most at risk of unplanned pregnancies, particularly women with a history of depression or who are experiencing intimate partner violence.

## Introduction

Globally an estimated eighty-five million pregnancies were unintended in 2012 [1]. Notwithstanding the fact that every unintended pregnancy represents a failure to meet the reproductive health needs of women and their partners, unintended pregnancies unnecessarily expose women to the risks associated with pregnancy, unsafe abortion and childbirth, thereby contributing to maternal mortality and morbidity. Unintended pregnancies have also been associated with adverse outcomes such as low birth weight, preterm birth, postnatal depression and neonatal mortality [2–11].

Most published data on the prevalence and determinants of unintended pregnancy come from the regular Demographic and Health Surveys (DHS) or studies using a similar methodology. These surveys ask women to think back up to five years to their last pregnancy and answer the question “At the time you became pregnant, did you want to become pregnant then, did you want to wait until later, or did you not want to have any (more) children at all?” This is used to categorise pregnancies as ‘intended’, if the woman says she wanted to get pregnant then, ‘mistimed’ if she wanted to get pregnant later, or ‘unwanted’ if she did not want to get pregnant at all. ‘Mistimed’ and ‘unwanted’ pregnancies are combined to form the ‘unintended’ categorisation. Whilst this has provided useful information, there are several limitations to this approach. Firstly, given the elapsed time between the pregnancy and data collection (up to five years after birth) there is considerable potential for recall bias. Secondly, data have shown that the same pregnancy is generally more likely to be reported as intended over time [12, 13]. Thirdly, the outcome of the pregnancy may influence the reported intention [14, 15]. Taken together this means that assessing intention in this way is likely to lead to an underestimate of unintended pregnancies.

Furthermore, dichotomizing all pregnancies is an over-simplification of the complex construct of pregnancy intention and may result in misclassification. To address this the London Measure of Unplanned Pregnancy (LMUP), a psychometrically validated measure of pregnancy intention, has been developed [16, 17]. It uses six questions, covering different aspects of the concept of pregnancy planning, to grade the pregnancy according to the degree of intention from zero to 12 (with zero being a completely unplanned pregnancy and 12 being a highly planned pregnancy).

Several studies have recently reported on the prevalence of unintended pregnancy and its correlates in a selection of low- and middle-income countries [18–28]. However, their findings have been inconsistent, as summarised in Table 1, making it difficult to draw conclusions as to the important determinants in other settings. For example, in young women in Tanzania, Calvert et al. found that increasing age, lower educational level and being unmarried were associated with ever having experienced an unplanned pregnancy, as were some sexual behavior factors [19]. A study in Paraguay found similar relationships with age, education and marital status but also found that a greater number of previous births was associated with unplanned pregnancy though socio-economic status (SES) was not [20]. In Ethiopia unintended births were more common in women who were young, unmarried, higher SES, higher parity and who had less than secondary education [21]. However, education and SES were not associated with unintended pregnancy in Ikamari et al’s study in Nairobi, Kenya [18].

**Table 1:** Findings of multivariate analyses of the determinants of unintended pregnancy in LICs and LMICs.

Study	Factors in analysis (significant factors in bold)
**Beguy**, 2014, Kenya [25]. UIPs^*^ in women aged 15–22 in two slums. Logistic regression.	Age, slum, SES, religion, **currently in school, currently married**, ethnicity, relationship with first sex partner, **age at first sex**, used contraception at first sex, **parent(s) living at home.**
**Calvert**, 2013, Tanzania [19]. UIPs in women aged 15–30. Hierarchical logistic regression.	**Age**, ethnicity, religion, **education**, **occupation**, **marital status**, time away in the past year, **knowledge of: where to access condoms;** where to access free condoms; HIV/STI acquisition; pregnancy prevention, attitude towards sexual health, **age at first sex**, **number of partners**, ever use of modern contraception, casual or regular partner in last year.
**Ikamari**, 2013, Kenya. [18] UIPs in women aged 15–49 in slum and non-slum settings in Nairobi. Logistic regression.	In slum settings: **age**, SES, ethnicity, education, occupation, **marital status, parity and household size.** In non-slum settings: age, SES, **ethnicity**, education, **occupation, marital status**, parity and household size.
**Dixit**, 2012, India [22]. Case-control study of national level data, matched on village and woman’s age.	**Religion,** caste**, SES,** woman’s education, **partner’s education, ever use of modern contraception, sex of last child, sex composition of living children,** experience of child loss, **birth interval.**
**Eggleston**, 1999, Ecuador [28]. Logistic regression of national level data.	**Age, area of residence, SES**, education, **marital status, parity**, **used modern contraception before most recent pregnancy,** number of modern methods known.
**Eliason**, 2014, Ghana [23]. Pregnant women attending ANC. Logistic regression.	**Marital status, parity, partner lives in same house,** aware of modern contraception, **aware of traditional contraception**, ever use of traditional contraception.
**Hamdela**, 2012, Ethiopia [24]. Cross-sectional survey of married pregnant women. Logistic regression.	Age, education, **parity**, family size, **previous unintended pregnancy, desired number of children, husband’s desired number of children.**
**Mazharul**, 2004, Bangladesh [26]. Analysis of DHS data. Logistic regression.	**Age, area of residence, rural v urban, SES**, **education,** employed, **parity, age at first marriage, used modern method of contraception.**
**Melian**, 2013, Paraguay [20]. Analysis of DHS data. Logistic regression.	**Age, rural v urban,** SES, employed, **education, marital status, number of living children.**
**Sedgh**, 2006, Nigeria [27]. Cross-sectional survey of women aged 15–49. Logistic regression.	Age, **region, residence, religion, SES,** education, **marital status, parity, ever used contraception.**
**Tebekaw**, 2014, Ethiopia [21]. Analysis of DHS data of ever-experience of unwanted birth. Logistic regression.	**Age,** rural v urban, **religion, ethnicity, SES**, **education, employed, parity, marital status, household size, knowledge of contraception, use of contraception,** media exposure, **decision-making power**, history of abortion.

*UIPs are ‘unintended pregnancies’

While the relationship of age, marital status and parity to pregnancy intention is fairly consistent, factors such as education and SES are less clear-cut. There are several possible reasons for these differences. Firstly, it may be because studies looked at different sub-groups of women (e.g. young women or married women) and were conducted in range of settings (rural, urban, slums) in diverse countries where the determinants may genuinely be different. Secondly, it may be due to the limitations of the DHS-style methodology used, which may have introduced recall bias and misclassification. Thirdly, some studies may have missed important determinants meaning that they have not been able to fully describe the relationships between determinants of pregnancy intention or deal with residual confounding. For example, two studies did not collect data on parity [19, 25] and two others did not consider SES [23, 24]. Two studies also compared ever-experience of an unintended pregnancy with current socio-demographic factors, which may further obscure the relationships [19, 21]. Finally, most studies’ analysis included all variables simultaneously in their multiple regression models and the causal pathways and mediating factors were not explored.

Victora et al. introduced the use of a conceptual hierarchical model to guide the analysis of determinants of an outcome in epidemiological studies where there may be ‘complex hierarchical inter-relationships’ between variables. In this case, unlike the creation of a predictive model, deciding which factors to include is based not only on statistical significance but also on a conceptual framework that describes the theoretical hierarchy among the determinants. As Victora et al. explain, ‘Ultimately, most ill health…may be ascribed to poverty [often assessed by] variables such as family income, parental education or the number and type of household appliances. Such factors, however, rarely cause ill health directly and henceforth are referred to as distal determinants. These factors are most likely to act through a number of inter-related proximate determinants [that] may be sub-divided into groups which are inter-related in a hierarchical or parallel way’ (p225) [29]. Approaching the analysis in this way helps to ensure that the effect of distal determinants, such as SES, is recognised and is not reduced or eliminated through incorrectly adjusting for proximate factors (i.e. those that are closer to the outcome). Failure to do so, may lead to the conclusion that the distal determinants are not important, rather than identifying their contribution to unintended pregnancy and describing how they are mediated through the proximate factors. The only study to have attempted this so far is Calvert et al. [19]. However their study was limited by the lack of data on parity, a likely determinant of pregnancy intention, and the fact that current socio-demographic factors were being compared with lifetime, rather than current, experience of unplanned pregnancy.

It is important to note that a conceptual hierarchical model is not the same as a hierarchical, or multi-level, statistical regression model, though the two may be used simultaneously.

Policy makers and health service planners need information on who is at risk of an unintended pregnancy to design and deliver effective programmes to prevent them and / or reduce their consequences. Other than DHS data, with its aforementioned limitations, there are no such data available for Malawi. The aim of this research was to use a robust measure of pregnancy intention combined with an analysis informed by a conceptual hierarchical model to determine the prevalence and distribution of pregnancy intention and the determinants of unintended pregnancies in Mchinji District, Malawi to inform service planning. The choice of a validated measure of pregnancy intention, its application during pregnancy and the use of a conceptual hierarchical model to inform the analysis, make this analysis unique and set the standard for future studies of this type.

## Methods

### Study setting and design

Mchinji District is a rural district in the central region of Malawi. It borders Zambia and Mozambique and has a population of around 500 000 (local data, unpublished). Malawi has a prevalence of unintended pregnancy of 45% [30] but there are no data on variations either by district or by women’s characteristics. Previous research divided the district into 49 geographical areas of approximately equal population [31]; from this sampling frame a random sample of 25 areas were selected to take part in research into pregnancy intention and maternal and neonatal outcomes. Using the pre-existing district-wide surveillance system, all pregnant women that were identified and were aged 15 and over in these 25 areas between March and December 2013 were invited to participate.

4,244 interviews were conducted with pregnant women in Mchinji District. These data were collected as part of a study into the relationships between pregnancy intention and pregnancy outcome. For this study a sample size calculation was conducted assuming 41% of pregnancies would be unplanned [32], a prevalence of 15% for adverse pregnancy outcome, [30, 33–35] a relative risk of 1.25, for 80% power at the 0.05 significance level; we thereby estimated that 3,737 pregnancy outcomes were needed.

One of 25 trained data collectors visited the pregnant women at home and conducted a 20-minute interview using a questionnaire programmed using CommCare ODK software on a smartphone. Women were asked demographic and obstetric history questions considered to be potential determinants of pregnancy intention on the basis of previous literature. including questions about the father of the child and previous episodes of depression or intimate partner violence (IPV). The variables considered as potential determinants of pregnancy intention are shown in Table 2.

**Table 2:** Variables considered as potential determinants of pregnancy.

Variables considered as potential determinants of pregnancy	
Socio-economic status quintile	Previous episodes of depression
Woman's education	Intimate partner violence
Partner's education	Woman's age
Partner's age	Number of live children
Marital status	First birth
Living arrangements	Time since last birth
Geographical area	Distance to health facility
Religion	Gestation (months)
Tribe	

A principal components analysis (PCA) was conducted to generate an asset-based measure of socio-economic status (SES). In addition to ownership of assets such as a bicycle and radio, variables included in the PCA were household characteristics, such as floor and roof materials and household density, and access to water and sanitation facilities. The distribution of SES score from this PCA was grouped into quintiles to create an ordered categorical variable for SES.

Pregnancy intention was measured using the validated Chichewa version of the LMUP [36]. In the absence of a validated tool for assessing previous depression, we worked with experts in the field to devise four questions about previous periods of low mood or anhedonia and whether these lasted for more than two weeks. These were used to categorise women as to the extent of possible previous depression. Women who experienced both low mood and anhedonia for more than two weeks were considered most likely to have experienced previous depression. Those who experienced only one of these, or who experienced them for a period of less than two weeks, were considered less likely to have experienced previous depression and women who reported neither of these were the least likely to have experienced previous depression. IPV was assessed using the Abuse Assessment Screen [37]. This asks about experience of abuse ever, in the last year or while pregnant as well as experience of sexual abuse. GPS readings of the location of the interview were taken and were used to calculate the distance to the nearest health facility.

### Statistical analysis

To assess the determinants of pregnancy intention, each potential determinant was considered in a univariate linear regression analysis before developing a multiple regression model. Collinearity was examined prior to the selection of variables for inclusion in the hierarchical model selection process. Where variables were collinear (e.g. marital status and living arrangements) only one variable was included in the model selection process. Categorical variables were entered into the models as sets of dummy variables in the standard way. For example, socio-economic quintile had the poorest quintile as baseline and the effects of the other quintiles were assessed relative to the baseline.

Several regression models were considered and a linear regression model with robust standard errors was selected as a good fit for the data. A random effects model was used to account for the clustering of our participants within geographical areas. The conceptual hierarchical model was used to inform the creation of the final multiple linear regression model [29]. The aim of the model was to understand the effects of various potential determinants of unintended pregnancies.

All analyses were conducted in Stata version 13. The multiple linear regression models were created using the ‘reg’ command with the ‘robust’ suffix, with ‘xtreg’ used for the random effects models.

### Conceptual hierarchical model

**Fig 1:**
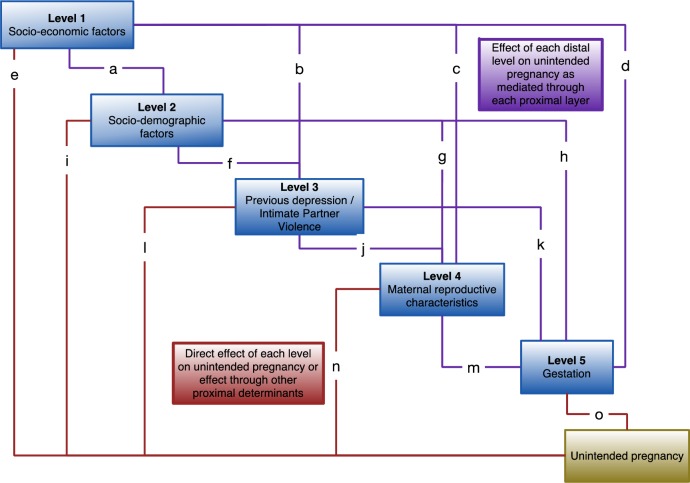
Conceptual hierarchical framework of risk factors for unintended pregnancy.

The conceptual hierarchical model (Fig 1) was developed based on the literature and temporal considerations with variables grouped into the five hierarchical levels as shown in Table 3. It starts with the most distal determinant, SES, as measured by the asset-based measure that had taken multiple variables into account in Level One at the top, and works down through increasingly more proximate determinants. Variables higher in the hierarchy influence those below them either indirectly, through their effect on the variables in other levels in the model, or directly. For example, SES may affect pregnancy intention indirectly through its effect on education (a Level Two variable, pathway a in Fig 1), previous depression (Level 3, pathway b), number of children (Level 4, pathway c) or gestation which is the most proximate determinant of pregnancy intention in our conceptual hierarchical model (Level 5, pathway d). SES may also have a direct effect on pregnancy intention (pathway e).

**Table 3:** Variables considered at each level of the hierarchical model.

Level of hierarchy	Variables considered
**Level 1—Socio-economic**	Asset index
**Level 2—Socio-demographic**	Woman's education
	Partner's education
	Partner's age
	Marital status
	Tribe
	Geographical area
**Level 3—Previous depression / IPV**	Previous episodes of depression
	Intimate partner violence
**Level 4—Maternal reproductive factors**	Woman's age
	Number of live children
	First birth / time since last birth
**Level 5—Gestation**	Gestation (months)

Socio-demographic variables were included in the second level of the hierarchy. Geographical area was included in Level Two, when it was introduced as a random effect to acknowledge the clustering of study participants within geographical areas. It was added at Level Two because areas do vary in their distribution of SES and we were only interested in the effect of area that was not due to differences in SES. Previous depression and intimate partner violence were introduced in Level Three, before maternal reproductive factors in Level Four, because we are looking at experience of these factors in the year prior to becoming pregnant and this may have played a role in becoming pregnant and the intendedness of this pregnancy. Gestation was included in Level Five as a marker of the time since conception and as the most proximate variable.

Although the development of a conceptual hierarchical model is based on the literature it is, nonetheless, subjective. It could be argued that educational achievement is a past exposure that determines current SES and should therefore be considered higher than SES, contrary to our hierarchy. Equally a high number of children may be a reason for previous episodes of depression rather than depression preceding a high number of children, as in our hierarchy. In recognition of this we conducted a number of sensitivity analysis to test the robustness of the findings to decisions we had made in constructing our conceptual hierarchical model.

### Application of conceptual hierarchical model to statistical analysis

Only variables that were associated with pregnancy intention at p<0.10 in the univariate analysis were considered for inclusion in the conceptual hierarchical model. Each variable or group of variables in a level was introduced simultaneously into the multiple regression model and the coefficients inspected. After each level was introduced, any of the new variables with p-values of > 0.10 were excluded in a manual backwards stepwise manner starting with the variable with the largest p-value. After each variable was excluded the significance of the remaining variables in the same level was examined. Once the removal of the variables was completed, since all remaining new variables in the level were p<0.10, the next level of variables was added to the regression model. Once a variable had been accepted into the model it was not subsequently removed, even if the inclusion of variables from lower levels in the conceptual hierarchical model resulted in it being no longer statistically significant.

The first regression model considers only the relationship between SES and pregnancy intention. This analysis shows the overall effect of the SES alone and not (improperly) controlled for the proximate factors that are partly determined by SES.

Level Two of the conceptual hierarchical model contains socio-demographic variables, such as education level and marital status, which are added to Model One. Level Two variables with a p-value of >0.10 were excluded using manual backwards stepwise regression to create Model Two. The coefficients for the remaining socio-demographic variables tell us their effect having (properly) controlled for SES. The new coefficients for the Level One variable of SES in Model Two give the estimate of its effect that is not mediated through the Level Two socio-demographic variables (pathway e).

The variables of Level Three in the conceptual hierarchical model were then added to the Model Two. Level Three contains previous depression and experience of IPV which are influenced by the factors in the Levels above and which can affect pregnancy intention either through Levels Four and Five (pathways j and k) or directly (pathway l). Level Three variables with p-values of >0.10 were excluded using manual backwards stepwise regression to create Model Three. The coefficients for the previous depression and remaining IPV variables in Model Three tell us their effect on pregnancy intention adjusted for the confounding roles of the socio-economic and socio-demographic variables in Levels One and Two. The new coefficient for the Level One variable, SES, gives an estimate of its effect that is not mediated through socio-demographic factors, previous experience of depression or IPV (pathway e) and the new coefficients for the socio-demographic factors are estimates of their effects that are not mediated through previous experience of depression or IPV (pathway i).

Next the maternal reproductive characteristics of Level Four of the conceptual hierarchical model were simultaneously added to Model Three. Level Four contains factors such as the number of live children a woman has and the time since the last birth that are affected by the determinants distal to it in Levels One to Three. Maternal reproductive characteristics may influence pregnancy intention through the final level of the model, Level Five, (pathway m) or directly (pathway n). Level Four variables with p-values of >0.10 were excluded using manual backwards stepwise regression to create Model Four. The coefficients for the remaining maternal reproductive characteristics tell us the effect of each factor on pregnancy intention adjusted for the confounding effects of the variables in Levels One to Three. The new coefficients for the variables in Levels One to Three are estimates of their effect on pregnancy intention that are not mediated through the variables at the lower levels of the hierarchy (pathways e, i and l).

Finally, the Level Five variable of the conceptual hierarchical model, gestation, was added to the Model Four. Gestation is a marker of the time since conception, that is the time that we are primarily interested in, and was included to account for any possible differences in reported level of pregnancy intention that are due to the timing of the assessment. Gestation influences pregnancy intention through pathway o.

The complete model now tells us: the residual effect of socio-economic status on pregnancy intention that is not mediated through socio-demographic factors, previous depression, IPV, maternal reproductive characteristics or gestation (pathway e); the residual effect of socio-demographic variables on pregnancy intention that is not mediated through previous depression, IPV, maternal reproductive characteristics or gestation (pathway i); the residual effect of previous depression and IPV on pregnancy intention that is not mediated through maternal reproductive characteristics or gestation (pathway l); the residual effect of the maternal reproductive characteristics that is not mediated through gestation (pathway n) and the unconfounded effect of gestation on pregnancy intention (pathway o). The residual effects may be either direct effects or effects that are mediated through other determinants that are not included in the model.

### Ethical Approval

The University College London Research Ethics Committee and the College of Medicine Research Ethics Committee at the University of Malawi granted ethical approval for this research (approval numbers 3974/001 and P.03/12/1273 respectively). Ethical approval was given to include pregnant women aged 15 and over. Field workers were trained to assess competency for consent in those aged below 18; these women gave their own written consent, no proxy was used. All women gave written informed consent to participate, by thumbprint if necessary, after they had read the information sheet and/or had the study explained to them. The participants retained the information sheet and one copy of the signed consent form; a second copy of the signed consent form was stored in a lockable cabinet in the main study office. Both ethics committees approved this consent procedure. Local approval to conduct the research in Mchinji District was given by the District Health Officer and the District Executive Committee.

## Results

### Background Characteristics

Over 99% of eligible women chose to participate in the study, suggesting that the data are representative of the population of pregnant women in Mchinji District.

The socio-demographic characteristics of the 4,244 women interviewed, along with their partner’s age and education level as reported by the woman, are shown in Table 4. There were no missing data for the mothers, but data on the fathers, as reported by the women, had a varying amount of missing data as indicated in the table. Most women were married (92%), had no education or primary education only (86.3%) and were Christians from the Chewa tribe. The age of women spanned the full reproductive period from 15 to 49 years (median 24 years); the fathers were generally older than the women (median age 28 years).

**Table 4:** Socio-demographic characteristics of women and their partners.

	Mother		Father	
	Freq.	Percent	Freq.	Percent
**Age (yrs)**			**n = 4,071**	
15–19	1,018	24.0	143	3.50
20–24	1,226	28.9	1,128	27.7
25–29	951	22.4	1,000	24.6
30–34	618	14.6	779	19.1
35–39	311	7.30	550	13.5
40–49	120	2.80	426	10.5
≥50	0	0	45	1.11
Range (median)	15–49 (24)		15–71 (28)	
**Education (level)**			**n = 4,174**	
None	422	9.94	334	8.00
Primary (1-8yrs)	3,215	75.8	2,678	64.1
Secondary (9-12yrs)	597	14.1	1,144	27.4
Tertiary (≥13yrs)	10	0.24	18	0.43
**Marital status**			
Married	3,905	92.0		
Unmarried	339	8.0		
**Distance to nearest health facility (kms)**				
Average (standard deviation)		5.9 (3.0)		
Range		0.1–15.8		
**Religion**				
Catholic	1,985	46.8		
Other Christian	2,091	49.3		
Muslim	94	2.21		
Other	74	1.74		
**Tribe**				
Chewa	3,597	84.8		
Ngoni	281	6.62		
Senga	207	4.88		
Yao	92	2.17		
Other	67	1.58		

Women’s obstetric history is shown in Table 5. Including the current pregnancy, women reported up to 15 pregnancies (median 3) and 12 previous births (median 2), with 11.9% of women reporting at least one miscarriage. The highest number of living children was nine due to previous stillbirths (5.33% of women had experienced at least one stillbirth) and child deaths (which 19.7% of women had experienced at least once). Over a third (34.4%) of the women who were currently pregnant had given birth within the last 24 months although the median time since the last birth was three years. On the basis of the women’s report of their last menstrual period women were between two and nine months pregnant (median 6 months) when interviewed.

**Table 5:** Women’s obstetric history.

**Obstetric history**					
	Freq.	Percent.		Freq.	Percent.
**Number of pregnancies**			**Previous miscarriage**		
**First**	1,172	27.6	None	3,739	88.1
**2nd - 3rd**	1,402	33.0	1	391	9.21
**≥ 4**	1,671	39.4	≥ 2	114	2.67
**Range (mode, median)**	1–15 (1, 3)		Range (mode, median)	0–6 (0, 0)	
**Number of previous births (live and still)**			**Previous stillbirth**		
**None**	1,240	29.2	None	4,018	94.7
**1–2**	1,423	33.5	1	198	4.66
**≥ 3**	1,581	37.3	≥ 2	28	0.66
**Range (mode, median)**	0–12 (0, 2)		Range (mode, median)	0–4 (0, 0)	
**Time since last birth (n = 2,995)**			**Previous child death (any age)**		
**< 24 months**	1,029	34.4	None	3,409	80.3
**2–3 years**	884	29.5	1	601	14.2
**3–4 years**	536	17.9	≥ 2	234	5.50
**4–5 years**	270	9.02	Range (mode, median)	0–6 (0, 0)	
**> 5 years**	275	9.18			
**Range (mode, median)**	7–264 (24, 36)				
**Number of living children**			**Current gestation (months)**		
**None**	1,352	31.9	Range (mode, median)	2–9 (6, 6)	
**1**	850	20.0			
**2**	659	15.5			
**3**	568	13.4			
**4**	407	9.59			
**≥ 5**	408	9.61			
**Range (mode, median)**	0–9 (0, 1)				

Almost 30% of women had experienced possible symptoms of depression in the year prior to their current pregnancy and in almost half of these women the episode lasted for more than two weeks. Over a fifth of women (22.4%) had experienced some form of abuse in their life. In all cases their husband or partner was most likely to be the perpetrator.

**Fig 2:**
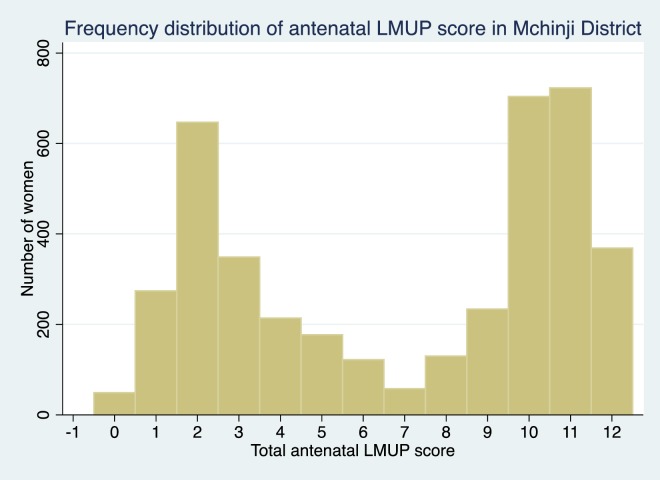
Frequency distribution of antenatal pregnancy intention as assessed on the LMUP.

The pregnant women interviewed in Mchinji reflected the full range of pregnancy intention with LMUP scores ranging from zero to 12. The bimodal distribution of intention is shown in Fig 2.

To estimate the prevalence of unintended pregnancy, cut-points have been suggested to divide the LMUP scores into categories of ‘unplanned’ (0–3 points), ‘ambivalent’ (4–9 points) and ‘planned’ (10–12 points) [16]. Using these cut-points 44.4% of pregnancies in Mchinji District were reported as planned; 32.6% were unplanned and women were ambivalent about the remaining 23.1%.

### Univariate analysis

The results of the univariate linear regression are shown in Table 6. Negative coefficients show that that characteristic is associated with a lower score on the LMUP, which indicates a less planned pregnancy. For example, the coefficient for an unmarried woman is -3.40; this means that unmarried women had an LMUP score that was 3.40 points lower than married women.

**Table 6:** Results of univariate analysis.

Univariate linear regression with robust standard errors			
Variable	β coefficient	95%CI	p-value
**Socio-economic status quintile**			
- Poorest	-	-	p<0.001
- Second-poorest	0.35	-0.03, 0.74	
- Middle	0.47	0.10, 0.85	
- Next-richest	0.74	0.35, 1.12	
- Richest	0.83	0.44, 1.22	
**Father’s age (years)**			
- 20–29	-	-	p<0.001
- 15–19	-2.02	-2.69, -1.35	
- ≥ 30	-0.98	-1.23, -0.73	
**Mother’s education level (yrs)**	0.15	0.11, 0.18	p<0.001
**Father’s education level (yrs)**	0.07	0.03, 0.10	p<0.001
**Unmarried**	-3.40	-3.89, -2.97	p<0.001
**Tribe**			
- Chewa	-	-	p<0.001
- Ngoni	-0.34	-0.84, 0.15	
- Senga	1.35	0.80, 1.90	
- Yao	0.26	-0.55, 1.06	
- Other	0.63	-0.37, 1.63	
**Previous depression**			
- none	-	-	p<0.001
- one/two for < 2 weeks	-0.95	-1.28, -0.61	
- one for ≥ 2 weeks	-1.93	-2.29, -1.57	
- both for ≥ 2 weeks	-2.23	-3.30, -1.16	
**Ever abused**	-1.04	-1.34, -0.74	p<0.001
**Abused in last year**	-1.61	-2.07, -1.16	p<0.001
**Abused while pregnant**	-1.17	-1.80, -0.55	p<0.001
**Sexual abuse**	-1.64	-2.55, -0.72	p<0.001
**Mother’s age (years)**			
- 18–29	-	-	p<0.001
- 15–17	-1.03	-1.46, -0.59	
- ≥ 30	-1.31	-1.59, -1.03	
**Number of live children**	-0.53	-0.60, -0.47	p<0.001
**First pregnancy**	1.43	1.17, 1.69	p<0.001
**Birth interval**			
- < 2 yrs	-	-	p<0.001
- 2-3yrs	1.44	1.10, 1.79	
- 3-4yrs	2.04	1.64, 2.44	
- 4-5yrs	2.70	2.19, 3.22	
- > 5yrs	2.55	2.04, 3.06	
**Gestation**	-0.10	-0.18, -0.02	P = 0.014

Women of higher SES reported their pregnancies as more planned (higher LMUP scores). Women whose partners were aged below 20 or above 30 tended to have lower LMUP scores (pregnancies that were less planned). Higher levels of maternal and partner education were both associated with pregnancies that were more planned. Women from the Senga tribe reported their pregnancies as more planned than women from other tribes. Previous experience of depression or IPV of any kind were associated with pregnancies that were more unplanned.

**Fig 3:**
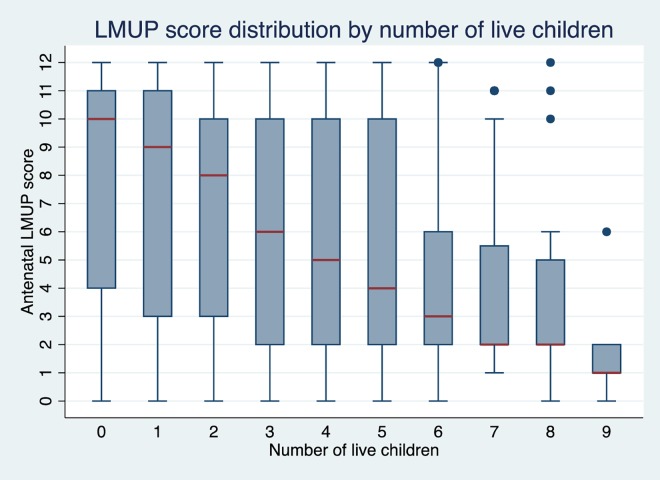
LMUP score distribution by number of live children.

Women aged below 18 or over 30 had lower LMUP scores than women aged 18–29. Each additional child a woman already had reduced the LMUP score for her current pregnancy by 0.53 points, making it more unplanned (shown in Fig 3). First pregnancies were reported as more planned than subsequent pregnancies, except in younger or unmarried women, and the longer the time since the last birth, the higher the level of planning reported for the current pregnancy.

The later in the woman’s pregnancy the interview took place, the more unintended they reported their pregnancy as. It may be that the reported intentions of women later in their pregnancy are influenced by concerns about the approaching birth. Alternatively it may be due to confounding by other factors, such as maternal age, marital status or education, if women who were visited later in pregnancy were different from those visited earlier. No significant associations were seen with religion (p = 0.225) or with distance to the health facility (p = 0.420) (data not shown). As living arrangements were highly correlated with marital status (99% of unmarried women were not living with their partner and 93% of married women were living with their partner all or most of the time) this variable was dropped in preference for marital status as this is a more commonly used variable in these analyses.

### Multivariable analysis

Table 7 shows the final versions of the multiple linear regression Models One to Four. To account for collinearity between the variables for first birth and time since last birth, one ordered categorical variable with women experiencing their first birth acting as the baseline and time since last birth grouped into less than two years, two to three years and more than three years. Tribe and father’s education were dropped from Model Two and ever-experience of abuse or abuse while pregnant were dropped from Model Three as they were not significant. Model Five is not shown as gestation, entered at the final level, was not significant and was therefore rejected, making Model Four the final regression model. Model Four describes the effects of factors at each level that are not mediated through factors at lower levels. The interpretation of this model is that, while increasing SES is associated with increasing pregnancy intention (as per Model One), the effect of SES is mediated through socio-demographic variables, previous experience of depression, abuse in the last year or sexual abuse and maternal reproductive factors. The socio-demographic factors of importance are marital status, partner’s age and mother’s education level, however the effect of mother’s education level appears to be mediated by maternal reproductive characteristics. Previous experience of depression and abuse in the last year are associated with lower pregnancy intention, even after controlling for SES and socio-demographic factors and independent of the effect that is mediated through maternal reproductive factors, though the effect of sexual abuse appears to be mediated by maternal reproductive factors. Mother’s age, number of live children and first birth / time since last birth are all associated with pregnancy intention after controlling for SES, socio-demographic factors and previous depression, abuse in the last year or sexual abuse

**Table 7:** Models One to Four of the multiple linear regression analysis based on a hierarchical approach.

		Model One				Model Two				Model Three				Model Four			
Level	Variable	β coeff	95%CI		p value	β coeff	95%CI		p value	β coeff	95%CI		p value	β coeff	95%CI		p value
1	Socio-economic status quintile																
	- poorest	-	-	-	-	-	-	-	-	-	-	-	-	-	-	-	-
	- second poorest	0.35	-0.03	0.74	0.002	-0.18	-0.63	0.27	0.00	-0.16	-0.64	0.31	0.77	-0.16	-0.62	0.30	0.00
	- middle	0.47	0.10	0.85		-0.21	-0.63	0.20		-0.22	-0.63	0.19		-0.20	-0.58	0.17	
	- next richest	0.74	0.35	1.12		-0.03	-0.46	0.41		-0.05	-0.52	0.42		0.12	-0.38	0.61	
	- richest	0.83	0.44	1.22		-0.11	-0.59	0.38		-0.06	-0.56	0.44		0.04	-0.46	0.54	
2	Mother’s education level (yrs)					0.10	0.07	0.14	<0.001	0.11	0.07	0.14	<0.001	-0.03	-0.06	0.01	0.12
	Father's age (years)																
	- 20–29					-	-	-	-	-	-	-	-	-	-	-	-
	- 15–19					-1.06	-1.67	-0.45	<0.001	-1.15	-1.75	-0.56	<0.001	-1.40	-1.98	-0.82	<0.001
	- ≥ 30					-0.94	-1.26	-0.63		-0.90	-1.19	-0.62		0.38	0.10	0.66	
	Unmarried					-3.45	-4.10	-2.80	<0.001	-3.27	-3.96	-2.58	<0.001	-3.62	-4.24	-3.00	<0.001
	Geographical area*					rho = 0.040				rho = 0.035				rho = 0.021			
3	Previous depression																
	- never									-	-	-	-	-	-	-	-
	- one/two < 2 weeks									-1.08	-1.49	-0.66	<0.001	-0.90	-1.31	-0.49	<0.001
	- one ≥2 weeks									-1.70	-2.43	-0.96		-1.34	-1.97	-0.70	
	- both ≥ 2 weeks									-2.07	-3.01	-1.14		-1.50	-2.43	-0.56	
	IPV—in last year									-1.03	-1.47	-0.58	<0.001	-0.83	-1.27	-0.3899422	<0.001
	IPV—sexual abuse									-0.86	-1.53	-0.18	0.01	-0.51	-1.18	0.15	0.13
4	Mother's age (years)																
	- 18–29													-	-	-	-
	- 15–17													-1.09	-1.48	-0.70	<0.001
	- ≥ 30													0.37	-0.01	0.74	
	Number of live children													-0.74	-0.87	-0.61	<0.001
	Birth interval																
	- first birth													-	-	-	-
	- < 24 months													-1.85	-2.21	-1.49	<0.001
	- 2–3 years													-0.59	-1.08	-0.11	
	- > 3 years													0.49	0.07	0.92	
r2 goodness of fit					0.0054				0.0766				0.1119				0.22

*rho is the proportion of variation explained by differences in geographical area, derived from the variability of random intercepts in a random effects model

From Model Four in Table 5 we can see that the most important determinant of pregnancy intention was marital status, as unmarried women have an LMUP score that is 3.62 points lower than married women with all other factors in the model held constant. Other key determinants, in descending order of importance, were a birth interval of less than two years, previous episodes of depression and young age of the father or mother.

We conducted two sensitivity analyses of the conceptual hierarchical model. The first assessed education above SES and the second assessed maternal reproductive factors above previous depression. Both resulted in the same final model.

While any woman can experience an unplanned pregnancy, inspection of the risk factors identified and consideration as to how these risks cluster led the to identification of three groups of women in Mchinji District who are at higher risk of unintended pregnancies. These were younger, unmarried women having their first pregnancy; older married women who have recently given birth and/or who already have as many children as they want; and women of any age, marital status or parity who have experienced depression, abuse in the last year or sexual abuse. The first two groups are mutually exclusive, however, the third group may overlap with either of the first two. Overall 38.6% of women fell into one or more of the high-risk groups. Of the women whose pregnancies were more unplanned (LMUP score ≤3 (32.6%, n = 1319)), just over half (51.1%) were in one of the three high-risk groups. Conversely 44.4% of all women (n = 1796) had a planned pregnancy (LMUP≥10) and of these, only 27.6% were in a high-risk group.

**Fig 4:**
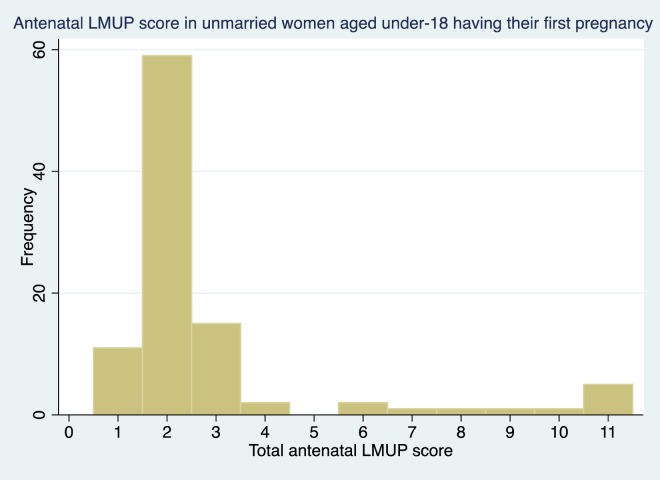
LMUP distribution in young, unmarried women having their first pregnancy

**Fig 5:**
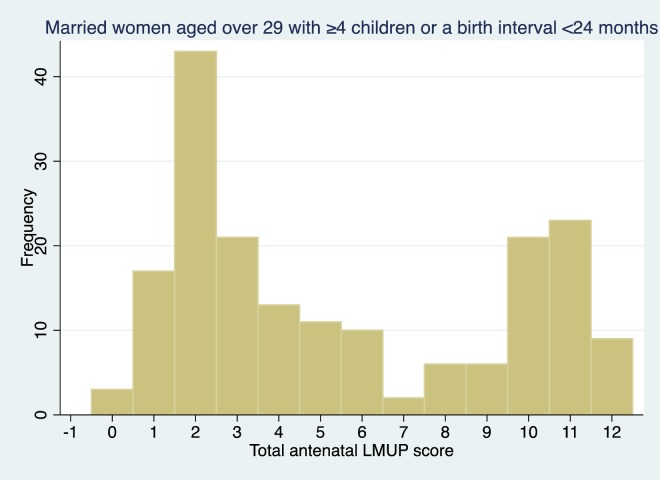
LMUP distribution in married women aged over-29 with at least four children or a birth interval of less than 24 months

**Fig 6:**
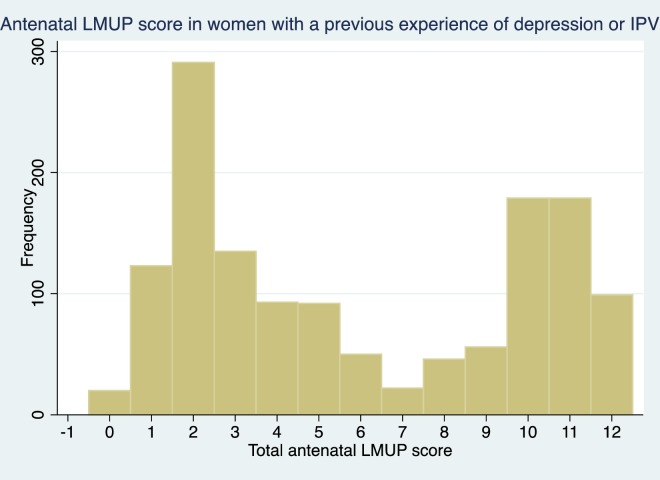
LMUP distribution of women with previous experience of depression, abuse in the last year or sexual abuse

The distributions of LMUP scores in these three groups is shown in Fig 4, Fig 5 and Fig 6 and are clearly markedly different to that of the general population shown in Fig 2 (p<0.001 on Mann-Whitney rank sum test for women in each of the at-risk groups compared to women who are in none of the groups).

## Discussion

The relationships between pregnancy intention and age, marital status and parity found in this study are in keeping with the findings of other studies [18–28]. Having used a conceptual hierarchical model to inform the analysis, this study is able to shed some light on the inconsistent findings of other studies with regard to SES and maternal education. Had we not used a conceptual hierarchical model, SES would not have been statistically significant and we would have concluded, like Ikamari et al [18] and Melian [20], that there was no relationship between SES and pregnancy intention. The same applies to maternal education, where our negative findings would have agreed with Ikamari et al [18]. However, in our univariate analysis, women with high levels of education reported their pregnancies as more planned and in the multiple linear regression the level of maternal education was significant in Models Two and Three. It was only once women’s age and reproductive factors were included in the regression model that maternal education was no longer statistically significant. This means that the effect of maternal education is mediated through these factors, not that it is unimportant. The same is true for SES; its effect is mediated through its influence on other factors. The differences in determinants seen between studies may therefore be due to the methodology of the analysis and/or whether or not the factors through which maternal education or SES affect pregnancy intention have been included.

### Strengths and Limitations

A strength of this research is the fact that pregnancy intention was assessed during pregnancy (as opposed to up to five years after the birth), reducing the risk of recall bias and removing the potential for the outcome of the pregnancy to influence the reported intention. Furthermore, we have assessed pregnancy intention using a psychometrically validated measure and have used the degree of intention in our analysis rather than grouping pregnancies into intended or unintended, something that has not been done before. Three recent studies using the LMUP to assess pregnancy intention did not use the whole scale in the analysis but instead classified pregnancies into two or three groups [38–40]. This results in a lot of lost information and only provides an estimate of the effect of pregnancy intention at one or two cut-points in the LMUP scale, rather than exploring the effect of each increase in the degree of pregnancy intention. Finally, we have used a conceptual hierarchical model to inform our analysis, allowing us to explore the direct and indirect ways in which factors contribute to pregnancy intention and enabling us to shed some light on the inconsistent findings of previous studies.

Further strengths are the community-based recruitment and high response rate (>99%). Whilst concerns could be raised about such a high level of consent, no material incentives to participate were offered and there were several factors that contributed to high acceptability and response rates. Firstly, the MaiMwana Project has worked within the community for over a decade and is well regarded locally for its work on improving maternal and child health. Secondly, this research was linked to the existing surveillance system, which the communities were already familiar with, and was introduced following a series of community sensitisation meetings and discussions with village chiefs and local traditional leaders who gave their permission for the research to be conducted in their areas. Finally, the data collectors were local women and they conducted recruitment face-to-face.

A limitation of this research is that early miscarriages and women who aborted their pregnancies were not included. In the study setting women do not disclose their pregnancy until the later stages and therefore women whose pregnancies were lost in the early months were not picked up by the surveillance system. Furthermore, abortion is illegal in Malawi. These factors may have led to an underestimate of the prevalence of unintended pregnancies. However, all studies will be limited to some extent by the difficulty of recognising early miscarriages.

Another limitation is that, while we have reduced the risk of recall bias by assessing intention during pregnancy, it is still a retrospective assessment of pregnancy intention as really we are interested in intention before conception. There is very little published data investigating how well women’s reported intentions during or after pregnancy match their pre-pregnancy intentions. This may be due, in part, to the significant practical problems facing this kind of research; it is necessary to recruit an extremely large cohort of non-pregnant women, assess their pregnancy intentions regularly (which may result in a Hawthorne effect) to capture their ‘real’ pre-pregnancy intentions, and follow them all up for long enough to detect sufficient pregnancies to power the study. Such prospective research is significantly hampered by the lack of a validated prospective measure of pregnancy intention. However, one recent study in Malawi compared seven different prospective and retrospective ways of measuring pregnancy intention [41]. When compared with each other they found that retrospective measures tended to overestimate levels of intended pregnancy and that prospective measures tended to underestimate intended pregnancies. At aggregate level there were no statistically significant differences between the measures, even between the ‘worst’–the retrospective post-birth assessment–and the ‘best’–a time varying prospective measure. The highest levels of agreement were seen between the last prospective measurement taken before conception and the first retrospective measure during pregnancy. Where pre-pregnancy assessments are not possible these data suggest that the assessment of intention during pregnancy is almost as good.

## Conclusion

More than half of all pregnancies in women in Mchinji District were reported as unplanned (scoring below 10 on the LMUP) making this an important public health problem. The implications of this for maternal and child health are significant; reducing unplanned pregnancies can save women’s and children’s lives as well as reduce pressure on health and other services.

To tackle this issue an integrated and multi-faceted approach to pregnancy planning and prevention needs to be taken. While all women and their partners should have access to services that help them to meet their reproductive health goals, there are several groups that should be targeted. Firstly, young women and men need to receive good quality, youth-friendly sexual and reproductive health services and education, whether or not they are married, to help prevent early, unplanned pregnancies. Secondly, to prevent rapid repeat pregnancies and additional children after desired family size is achieved, pregnant women should be counseled about post-partum family planning options during pregnancy, and services should be configured to provide contraception at delivery, at post-partum checks and at child health appointments according to the woman’s choices. While in the study setting there is a culture of abstinence for six weeks after birth, very often intercourse resumes before women restart contraception leading to unplanned pregnancies. For those women who would like to become pregnant again soon, or who are not able to use contraception for some reason, pre-conception advice and support should be given. Finally, particular attention needs to be given to women with a history of depression or who are experiencing IPV as they are at increased risk of unplanned pregnancies. Given the relationships between these factors and broader determinants of health, such as SES, and the general acceptance of IPV in Malawian culture [30], if not in legislation [42], individual level interventions should be supplemented with community-based programmes. This research also supports the importance of education, for both women and men, in improving health outcomes.

While this research has described the prevalence of unplanned pregnancy and its determinants in Mchinji District, further work needs to be done to assess the impact of pregnancy intention on pregnancy outcome.
